# Cell-free supernatant of *Bacillus velezensis* suppresses mycelial growth and reduces virulence of *Botrytis cinerea* by inducing oxidative stress

**DOI:** 10.3389/fmicb.2022.980022

**Published:** 2022-08-05

**Authors:** Huanlan Zhao, Kui Liu, Yezhen Fan, Jiacan Cao, Huanghuan Li, Wu Song, Yongsheng Liu, Min Miao

**Affiliations:** ^1^School of Food and Biological Engineering, Hefei University of Technology, Hefei, China; ^2^Institute of Botany, Chinese Academy of Sciencess, Beijing, China; ^3^School of Food Science and Technology, Jiangnan University, Wuxi, China; ^4^School of Biological Science and Engineering, North Minzu University, Yinchuan, China; ^5^Ministry of Education Key Laboratory for Bio-Resource and Eco-Environment, State Key Laboratory of Hydraulics and Mountain River Engineering, College of Life Science, Sichuan University, Chengdu, China; ^6^School of Horticulture, Anhui Agricultural University, Hefei, China

**Keywords:** *Botrytis cinerea*, *Bacillus velezensis*, cell-free supernatant, reactive oxygen species, damage of cell membrane

## Abstract

As a notorious pathogenic fungus, *Botrytis cinerea* has been reported to infect more than 1400 species of plants and cause postharvest gray mold of numerous economic fruits, leading to substantial economic losses. Traditional chemical fungicides in pathogen control have potential issues regarding environmental pollution, disease resistance and human health. More safety and efficacious prevention technique of postharvest gray mold are in urgent demand. This study aims to investigate the potential function and mechanism of *Bacillus velezensis* to control gray mold for harvested fruits. The results showed that the cell-free supernatant (CFS) generated from *B. velezensis* strain A4 was able to inhibit spore germination, germ tube elongation and hyphal growth of *B. cinerea in vitro*, and impair the pathogenicity of *B. cinerea* on the four tested fruits. Further analysis demonstrated that CFS significantly reduced the expression of genes associated with growth and pathogenicity and weakened the ability of *B. cinerea* spores to penetrate plant cell walls in a dose-dependent manner. Moreover, the CFS destroyed the membrane of hyphae, resulting in exosmosis of cell contents and caused hyphal cells to accumulate excessive reactive oxygen species (ROS), leading to hyphal oxidative damage. Our findings indicate that *B. velezensis* CFS can damage *B. cinerea* mycelial cells by promoting excessive accumulation of ROS to realize its biological control function.

## Introduction

Fruits provide higher percentages of vitamins, minerals, and dietary fiber, which give many health benefits and significant economic value (Li et al., 2019a; Shen et al., 2019). Particularly vulnerable to the proliferation of various pathogens due to the soft texture, susceptibility to mechanical injury, and high water content (Yang et al., 2010; Xu et al., 2019). The prevailing fungal rot has been considered as one of the most severe postharvest diseases, with an average incidence of 30-50% during packing, storage and transportation. This devastating fruit decay is associated with a variety of pathogenic fungi, mainly including *Botrytis cinerea* (Diao et al., 2020; Ma et al., 2022), *Penicillium expansum* (He et al., 2019), *Fusarium* spp. (Peng et al., 2022), *Phytophthora* (Adaskaveg et al., 2015), and *Alternaria alternate* (Yan et al., 2015).

Numerous studies showed that gray mold caused by *B. cinerea* is one of the most severe diseases that reduce the yield and quality of fruit (Wang et al., 2021b). *B. cinerea* possesses an abroad host range and can infect more than 1400 plants, including tomato, strawberry, apple, kiwifruit and grape (Sansone et al., 2005; Li et al., 2019c; Kai et al., 2020; Galli et al., 2021; Niu et al., 2021). Over the past few decades, the application of chemical fungicides has been widely used to control gray mold disease (Gao et al., 2018). However, increasing concerns about potential toxicity and environmental risks from chemical fungicide residues have driven the exploration of alternative approaches to inhibit the pathogen (Ji-Chen et al., 2013). New measures such as biological control with antagonistic microorganisms and natural compounds have been demonstrated as a promising tool in controlling the gray mold and reducing the detrimental effects of fungicides (Droby et al., 2009; Lu et al., 2021).

Biocontrol bacteria and yeast play an important role in the biological control of gray mold (Dal Bello et al., 2008; Chaouachi et al., 2021). Nutritional and spatial competition have been shown as the main antagonistic mechanisms of biocontrol agent against pathogens, but the effect of a biocontrol agent is easily affected by the environment (Zhang et al., 2020). *Bacillus* is one of the bacteria widely distributed in soil and rhizosphere as well as on the surface of plants, with a robust antagonistic ability against various pathogenic fungi on multiple fruits (Han et al., 2016). Therefore, it has increasingly been used to control postharvest disease (Fira et al., 2018). A vast array of secondary metabolites with antagonistic activities produced by *Bacillus* plays a significant role in biocontrol against postharvest diseases. Previous study (Arroyave-Toro et al., 2017) showed that *B. Subtilis* EA-CB0015 cells and its CFS inhibited the growth of the nine fungal pathogens with different susceptibilities *in vitro*. In addition, the filtrate of *B. amyloliquefaciens* BA17 exhibited a potent antifungal effect against *B. cinerea* and significantly reduced spore germination, spore production, and hyphal growth of *B. cinerea*, thus being considered as a suitable biocontrol agent for the harvested green beans (Li et al., 2020). However, as a potential biocontrol bacterium, the antagonistic mechanism of *B. velezensis* against pathogens needs further investigation.

## Materials and methods

### The resuscitation of bacillus velezensis strain A4 and preparation of its cell-free supernatant

*Bacillus velezensis* strain A4, previously isolated and identified in our laboratory, was activated on the Luria-Bertani (LB) plate at 28°C for 16–24 h. The single colony was cultured until the number reached 6 × 10^8^ CFU/mL in a shaker flask at 28°C and transferred to the deodorizing fermentation medium (LB liquid medium containing 2% glucose) with 0.5% inoculum and incubated at 28°C for 72 h (180 rpm). Then, the culture was filtered through a bacterial filter to obtain CFS as described previously (Li et al., 2020). The CFS was diluted to 2%, 4% and 6% of the final working volume fraction.

### Preparation of *Botrytis cinerea* and its spore suspension

*Botrytis cinerea* was kindly provided by professor Danfeng Zhang of the Hefei University of Technology. It was cultured on potato dextrose agar (PDA) plates for 7–10 d at 23 ± 2°C. Spores were harvested by immersing the culture surface with sterile distilled water and followed by filtered with four layers of sterile cheesecloth. The number of resulting spores suspension was determined using a hemocytometer, and the final concentration was adjusted to 1 × 10^6^ spores/mL with sterile water (Ji et al., 2018).

### Fruits

All the fruits (cherry tomato, apple, kiwifruit and strawberry) with suitable maturity used in the experiment were purchased from supermarkets. The fruits were uniform in size, free of disease infection and physical injury, disinfected with 2% (v/v) sodium hypochlorite for 2 min, rinsed with tap water, and air-dried at room temperature (Cui et al., 2021).

### Antifungal experiment of cell-free supernatant *in vitro*

Ten micro liter of spore suspension of *B. cinerea* was added to the center of a petri dish containing 20 mL of PDA that was amended with CFS of A4 at each working volume fraction, and further incubated under 25°C. PDA plates without CFS were used as a control. The colony diameter was determined and inhibition rate was calculated (Wang et al., 2020). Each treatment consisted of five plates, and the experiment was conducted three times. Additionally, a mycelial plug (0.7 cm diameter) of *B. cinerea* was incubated in potato dextrose broth (PDB) containing different working volume fractions (0, 2, 4 and 6%) of A4 CFS at 25°C for 72 h with shaking (180 rpm). Then, the weight of mycelia was measured after collecting and drying.

### Effects of A4 cell-free supernatant on controlling gray mold caused by *Botrytis cinerea* in post-harvest fruits

Cherry tomato in this assay was divided into four groups comprising at least 80 fruits in each group and was punctured with the sterile stainless needles at the two equatorial sides of each fruit to form two symmetrically uniform holes (3 mm deep and 3 mm wide). Then, 1 μL A4 CFS containing different working volume fractions (0, 5, 10, and 15%) with 1 μL spore suspension of *B. cinerea* at 1 × 10^6^ spores/mL were inoculated in the pretreated holes. All inoculated fruits were air-dried at room temperature and maintained a 90–95% relative humidity (RH) at 25°C for disease development. The assays of A4 CFS treatment on *B. cinerea* in apple, kiwifruit and strawberry were performed similarly as described above. The difference is that the depth and width of the inoculation point are about 5 mm, respectively. Meanwhile, the total volume of inoculation was adopted to 10 μL, and the *B. cinerea* spore suspension concentration was 1 × 10^5^ spores/mL.

### Effect of cell-free supernatant on spore germination of *Botrytis cinerea*

About 1.5 × 2.5 cm thin slice was cut from the PDA plate containing 0%, 2%, 4% or 6% CFS using a sterilization blade and affixed on a sterile slide. Then 40 μL of 1 × 10^6^ spores/mL spore suspension was spread evenly on the slice and then cultured in a 25°C incubator. Spores germination and germ tube elongation were observed at 0, 3, 6, 9, and 12 h. The spores (approximately 200) were randomly observed to calculate the germination rate and measure the germ tube length in each treatment (Liu et al., 2017).

### Effect of cell-free supernatant on the ability of *Botrytis cinerea* to penetrate onion cell and cherry tomato fruit

The effect of CFS on the ability of *B. cinerea* hyphae to penetrate cell walls of onion was evaluated as described (Hua et al., 2019). CFS was added to 1 × 10^6^ spores/mL spore suspension to obtain a final working volume fraction of 0, 2, 4, and 6%. 200 μL of the above spore suspension were placed and incubated on the onion epidermal peels in a petri dish. Hyphae penetration into onion cells was stained with 0.25% trypan blue and observed under a light microscope after 2 and 4 days.

The tomato fruits were treated with 0% or 15% CFS and inoculated with *B. cinerea* for 24 h in method 2.5 as materials for the electron microscope analysis. The sample (1 cm deep × 1 cm wide) was harvested from the inoculation site, fixed with 2.5% glutaraldehyde solution, dehydrated with ethanol gradient, and freeze-dried as described (Rizwana et al., 2021). The dried samples were observed by scanning electron microscopy (Hitachi S-4800; Hitachi, Japan).

### Relative expression levels of genes associated with growth and pathogenicity in *Botrytis cinerea*

The 7 mm-diameter mycelial disks of *B. cinerea* were incubated in PDB at 25°C for 72 h with shaking (180 rpm). Then the CFS was added to the final working volume fraction of 0, 2, 4, and 6%. Mycelia were collected at 24 h after incubation with the CFS, washed twice by phosphate buffer, and stored at 4°C and –80°C until further processing. Total RNA of *B. cinerea* was extracted using Trizol reagent (Tiangen, China) from the mycelia. The cDNA was obtained using a PrimeScript RT reagent kit (TaKaRa, Dalian). RT-qPCR analysis was performed using a Step One Plus Real-Time PCR System (Applied Biosystems) with SYBR premix ex Taq (TaKaRa, Dalian). Primer sequences, PCR sections and analysis method were applied as described previously (Wang et al., 2020, 2021c). Each treatment was composed of three biological replicates, and the experiment was conducted twice.

### Effect of cell-free supernatant on mycelial morphology and microstructure of *Botrytis cinerea*

The mycelia were collected and processed according to the morphology and microstructure described in the method section “Effect of CFS on the ability of B. cinerea to penetrate onion cell and cherry tomato fruit” and “Relative expression levels of genes associated with growth and pathogenicity in *B. cinerea*.” It was observed by laser scanning confocal microscope or scanning electron microscopy (SEM).

### Determination of membrane integrity

A moderate amount of hyphae processed in section “Relative expression levels of genes associated with growth and pathogenicity in *B. cinerea*” were immersed in 5 mM propidium iodide (PI) and then incubated in a 30°C water bath for 20 min. Then, the stained hyphae were washed three times with a phosphate-buffered saline (PBS) buffer (0.05 M, pH 7.0) to remove the dye. The giving red fluorescence was observed using confocal microscopy.

### Malondialdehyde content and cytoplasmic content leakage detection

The mycelia samples at –80°C were well ground into powder using liquid nitrogen for MDA assay. The content of malondialdehyde (MDA) was determined by the thiobarbituric acid (TBA) method (Jiao et al., 2020).

Nucleic acid and protein leakages were determined as described with some modifications (Bradford, 1976; Cai et al., 2015). The 72 h of cultured mycelia were pooled and washed with sterile distilled water. Subsequently, 0.5 g mycelia were suspended in 0%, 2%, 4%, and 6% CFS and cultured in a shaker at 25°C for 0, 3, 6, 9, and 12 h. The resulting cultures were filtrated and used to determine nucleic acid and soluble protein concentration.

### Detection of reactive oxygen species accumulation and relative expression levels of producing genes

Hyphae in method section “Relative expression levels of genes associated with growth and pathogenicity in *B. cinerea*” were stained with 10 μM 2′,7′-dichlorodihydrofluorescein diacetate (H_2_DCFDA; Invitrogen, Eugene, OR, United States), and the H_2_O_2_ accumulation was observed using confocal laser microscopy (LSM800; Zeiss, Oberkochen, Germany) as the description (Cui et al., 2021).

Total RNA extraction and qRT-qPCR test process are the same as in method section “Relative expression levels of genes associated with growth and pathogenicity in *B. cinerea*” and the primers information are shown in Table 1.

**TABLE 1:** Relevant information of *NoxA* and *NoxB.*

Gene name	Accession number	Primer name	Primer sequences (5′→3′)
*NoxA*	AM900412.1	*BcNoxA*-F1	CCCAAATTGTTAGAGTCGTTCG
		*BcNoxA*-R1	GATGTGATGGTGAATGGATGC
*NoxB*	AM900413.1	*BcNoxB*-F1	TTGATGTCTGGTGAGATGACG
		*BcNoxB*-R1	CATGGAGTAAAGCGAAAACGAAG
*Actin*	XM001553318	*BcActin*2F	GGCCTGTGCTAATCATCGGT
		*BcActin*2R	TGGGAGTTGTTGTTTTGCTCG

### Assay of enzyme activity

The mycelia sample was well ground into powder with liquid nitrogen. 0.5 g powder sample was homogenized in ice-cold extraction buffer [100 mM sodium phosphate buffer pH 7.0, 5% polyvinyl pyrrolidone (PVPP), 1% Triton X-100 and 1 mM EDTA] for crude enzymes solution. The homogenates solution was centrifuged at 12,000 g for 20 min at 4 °C, and the supernatant was collected for subsequent determination of enzyme activity. The enzyme activities of SOD, POD and CAT were detected using a commercial kit (Jiancheng, Nanjing) according to the manufacturer’s instructions, respectively.

### Effect of n-acetylcysteine on the recovery of *Botrytis cinerea* with cell-free supernatant

The process of hyphal culture is the same as in method section “Relative expression levels of genes associated with growth and pathogenicity in *B. cinerea*”. Meanwhile, NAC with a final concentration of 10 mM was added to the treatment group additionally, and the hyphae were stained with H_2_DCFDA as in section “Detection of ROS accumulation and relative expression levels of producing genes.” The experimental process of NAC recovering CFS affecting mycelia growth is similar to method section “Antifungal experiment of CFS *in vitro*.” Specifically, NAC was added to a final concentration of 10 mM in the treatment group, and an equal amount of sterile water was added to the control group. After 72 h of culture, all the mycelia were collected and dried to constant weight.

### Statistical analysis

The statistical analyses were conducted using SPSS 23.0. Results data were analyzed using a one-way analysis of variance (ANOVA) and shown as the mean ± standard errors of least squares means (SEM). According to Duncan’s multiple range tests, a p-value of <0.05 was accepted as statistically significant. The significant difference was marked by letter, and different letters indicated the significant difference among the treatments.

## Results

### The cell-free supernatant of *Bacillus velezensis* retards vegetative growth of *Botrytis cinerea* and impairs its pathogenicity on harvested fruit

**FIGURE 1:**
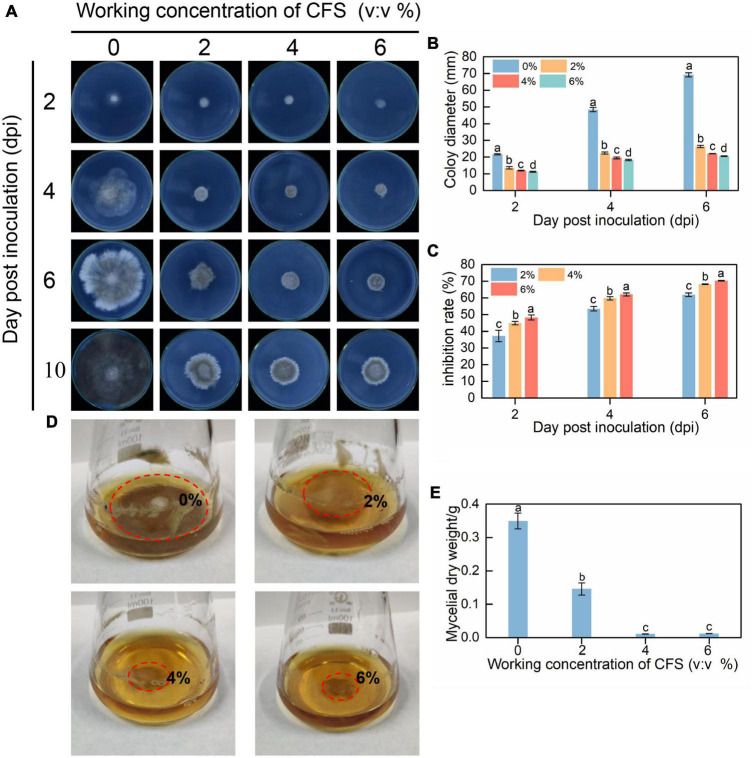
The cell-free supernatant (CFS) of *Bacillus velezensis* inhibited vegetative growth of *Botrytis cinerea* both on PDA (potato dextrose agar) plate **(A–C)** and in PDB (potato dextrose broth) medium **(D,E)**. **(A)** Images were recorded after 2, 4, 6, and 10 days of incubation. **(B)** Statistical analysis for colony diameters. **(C)** The inhibition rate of CFS of different concentrations. **(D)** Images were recorded after 3 days of incubation, and the red circles represent the mycelia ball in different concentrations of CFS. **(E)** Statistical analysis for mycelial dry weight. The experiments were conducted in triplicate, and the data shown are means ± standard deviations (*n* = 5). According to Duncan’s multiple range test, columns marked by different letters represented statistically different. (*p* < 0.05).

The inhibitory effect of CFS on the vegetative growth of *B. cinerea in vitro* was observed on PDA plate and PDB liquid medium. Equivalent amounts of spores and mycelial disks of *B. cinerea* were inoculated into a PDA plate and a PDB liquid medium containing different concentrations of CFS, respectively. As shown in Figures 1A–C, CFS suppressed the mycelial growth of *B. cinerea* on the PDA plate in a concentration-dependent manner. After 6 days of incubation, the colony diameter in the 6% CFS treatment was only about 30% of that in the control group. In addition, after 10 days of incubation, the mycelia fully covered the control dish and turned black, while the outer edge of the mycelia was white in all CFS treatment groups. Meanwhile, the growth rate of *B. cinerea* in the PDB medium was also inhibited by CFS in a dose-dependent manner. We observed that the mycelial ball in the control group was larger than that in the CFS treatment group, and the size of the mycelial ball in the treatment group was negatively associated with the CFS concentration (Figure 1D). When the volume fraction of CFS reached 6%, the dry weight of mycelia was only 3% of that in the control group (Figure 1E). These *in vitro* results indicated that CFS of *B. velezensis* had potent antimicrobial activity on *B. cinerea*.

**FIGURE 2:**
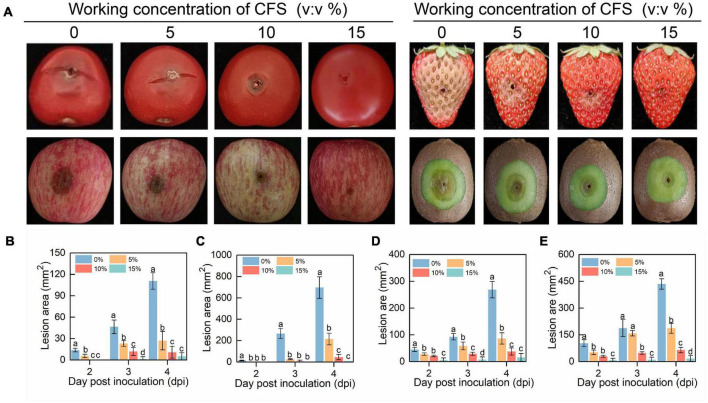
The efficacy of cell-free supernatant (CFS) on pathogenicity of *Botrytis cinerea* on postharvest fruits. **(A)** Photographs of diseased fruits were taken 4 days after treatment. Lesion area on harvested cherry tomato **(B)**, strawberry **(C)**, apple fruit **(D)** and kiwifruit **(E)** were measured by using Image J. Histograms with different letters at each time point indicate significant differences according to Duncan’s multiple range tests at *p* < 0.05.

To test the effect of CFS on the virulence of *B. cinerea* in harvested fruits which are prone to gray mold infection caused by *B. cinerea*, cherry tomato, strawberry, apple and kiwifruit were examined following *B. cinerea* inoculation. In agreement with the results from the *in vitro* inhibition assay, CFS strikingly reduced the lesion area of *B. cinerea* on all the tested fruits in a dose-dependent manner (Figure 2). These results showed that the CFS of *B. velezensis* strain A4 inhibited the vegetative growth of *B. cinerea*, and significantly alleviated the pathogenicity of *B. cinerea* on different fruits, indicating that the strain A4 had the potential to control the postharvest gray mold.

### The cell-free supernatant inhibits spore germination and germ tube elongation

**FIGURE 3:**
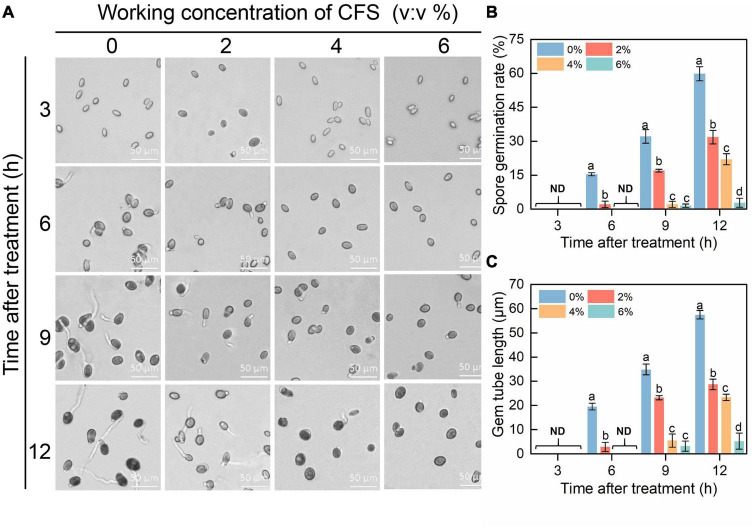
The efficacy of cell-free supernatant (CFS) on spore germination and germ tube elongation of *Botrytis cinerea*. **(A)** Representative images of each treatment were recorded after inoculation every 3 h until 12 h. **(B,C)** Statistical analysis for spore germination **(B)** and germ tube elongation **(C)**. About 200 spores were randomly calculated in each experiment by ImageJ, and the statistical analysis was performed on triplicates of 200 spores. The standard error of the mean value is represented by a vertical bar. Columns with different letters indicated significant differences on the basis of Duncan’s multiple range test (*p* < 0.05).

In order to explore the possible mechanism of CFS, the effects of CFS on spore germination and germ tube elongation of *B. cinerea* were examined with a microscope. The results showed that spore germination and germ tube elongation of *B. cinerea* were obviously inhibited in the presence of CFS, and the inhibitory effects were positively correlated with the CFS concentration. The decreased spore germination rate was observed upon increased CFS concentration (Figures 3A,B). The germ tube length was reduced by 50.1, 59.4, or 90.9% by adding CFS at a final volume fraction of 2%, 4%, or 6%, respectively, after 12 h treatment (Figures 3A,C). The results showed that CFS could prevent the pathogen *B. cinerea* via inhibiting spore germination and germ tube elongation.

### The cell-free supernatant inhibits the ability of *Botrytis cinerea* to penetrate onion cells and cherry tomato fruit

**FIGURE 4:**
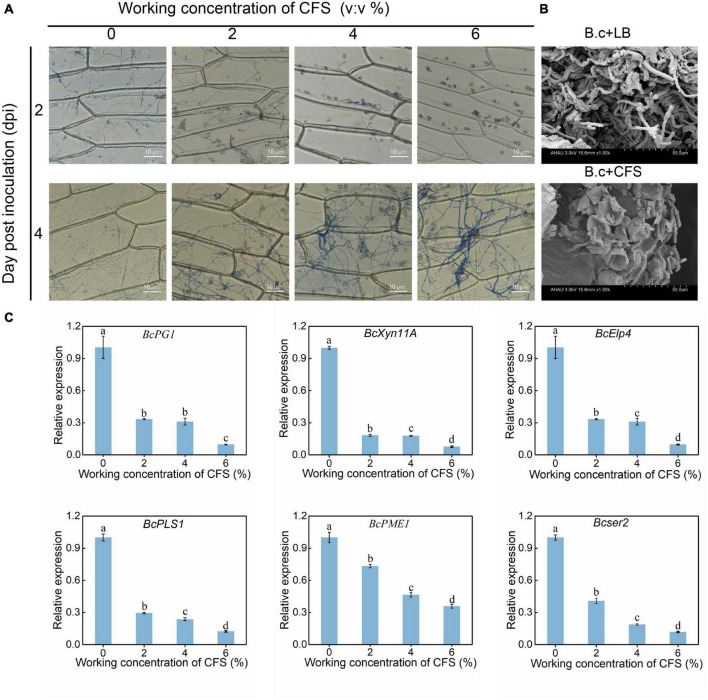
Effect of cell-free supernatant (CFS) on the ability of *Botrytis cinerea* to penetrate onion cells and cherry tomato fruit. **(A)** Onion epidermal peels were cut into 1.5 × 1.5 cm pieces, then were treated with different concentrations of CFS and inoculated with 200 μL of *B. cinerea* spore suspension. Images were recorded after inoculation for 2 and 4 days using a microscope. **(B)** The situ status of *B. cinerea* spores in tomatoes was detected by scanning electron microscopy (SEM) after 24 h treatment. B.c + LB and B.c + CFS represent that tomatoes inoculation 1 μL LB liquid medium or cell-free supernatant and subsequently inoculated with 1 μL spore suspension *B. cinerea* spore suspension (1 × 10^6^ spores/mL), respectively. **(C)** RT-qPCR analysis of critical genes reportedly involved in regulating the growth or pathogenicity of *B. cinerea.* The different letters (*p* < 0.05) within the same time point denotes a significant difference among several reported genes.

The ability of *B. cinerea* hyphae to penetrate the host cell walls was further investigated using epidermal cells of onion and tomato. As shown in Figure 3A, Two days after the inoculation, more spores of *B. cinerea* were found on the upper surface of the onion epidermis in the treatment groups, while spores had been germination into the control group (Figure 4A). Four days after inoculation, the hyphae of the control group had passed through the cell wall and entered the interior of the onion epidermis. The hyphae in treatment groups were dyed blue by trypan blue, indicating that the hyphae had lost vitality (Figure 4A). Meanwhile, scanning electron microscopy observation found that the spores of *B. cinerea* treated by CFS hardly germinate into hyphae on cherry tomato fruits, and some even had apoptotic symptoms. Contrary to the CFS treatment, spores in the control condition germinated into hyphae and covered the fruit surface typically (Figure 4B). The penetration of hyphae into the host epidermis has been considered a crucial step for pathogen infection, resulting from multiple gene regulations (Mendgen and Deising, 1993; Leroch et al., 2015). Combined with all of the above results, several previously reported genes (*BcPG1*, *BcXyn11A*, *BcElp4*, *BcPLS1*, *BcPME1*, and *Bcser2*) related to the growth or pathogenicity of *B. cinerea* have been detected by qRT-PCR, and their expression levels were indeed down-regulated after CFS application in a dose-dependent manner (Figure 4C). The results state that A4 CFS played a role in inhibiting the expression of genes regulating hyphal penetration, resulting in the decline of hyphal penetrability and pathogenicity of *B. cinerea.*

### The cell-free supernatant damages and ruptures the hyphae of *Botrytis cinerea*

**FIGURE 5:**
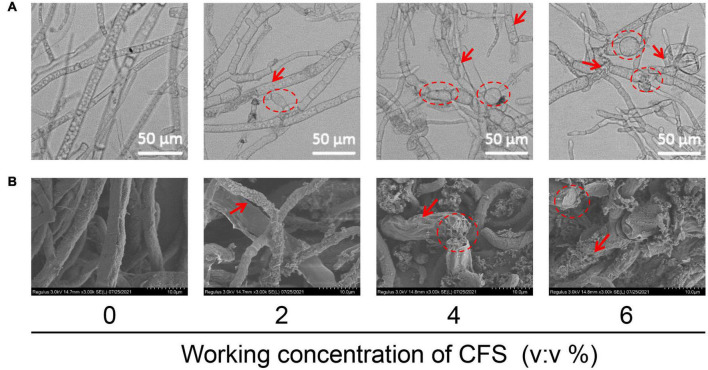
Effects of cell-free supernatant (CFS) on the morphological structure of *Botrytis cinerea*. **(A)** Effects of CFS on the morphological structure of *B. cinerea* were tested in a PDB medium containing a different working concentration of CFS. The hyphae were collected after 24 h of treatment and images were recorded using the microscope. **(B)** Scanning electron micrographs of *B. cinerea* treated with CFS at different working concentrations.

In addition to the effect of CFS on hyphal penetration, the mechanism of CFS on inhibiting hyphal growth currently remains elusive. Therefore, we explored whether CFS affected the hyphae morphology of *B. cinerea* leading to this inhibition. As shown in Figure 5A, When exposed to CFS, the hyphae surface was rough and unsmooth (the red arrow) and hyphae were swollen and deformed (the red circle) as compared with the control group. Then, the scanning electron microscope analysis further proved that the CFS roughened the hyphae (see the red arrow in Figure 5B). In addition, 4% and 6% of CFS destroyed the integrity of hyphae (the red circle in Figure 5B), while the control hyphae were intact (Figure 5B). These observations indicated that CFS could damage the hyphal morphology of *B. cinerea* in a dose-dependent manner.

### The cell-free supernatant damages hyphal cytomembrane by causing peroxidation in *Botrytis cinerea*

**FIGURE 6:**
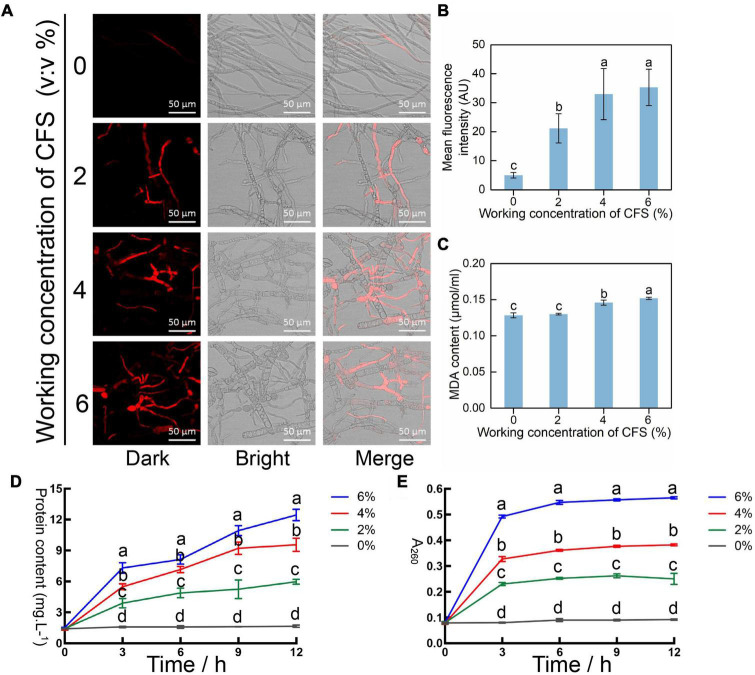
Injury efficacy of cell-free supernatant (CFS) on the hyphal cytomembrane of *Botrytis cinerea in vitro*. **(A)** Detecting the integrity of membrane of hyphal cells at 24 h after the application of CFS was stained by propidium iodide (PI). The fluorescence signal was detected using a laser scanning confocal microscope at 405 nm excitation and 633 nm emission. **(B)** The relative intensity of fluorescence of each treatment was measured by ImageJ. **(C)** Malondialdehyde (MDA) content which refers to the degree of membrane lipid peroxidation of hyphal cells, was recorded after treatment with CFS for 24 h. **(D)** Total protein content in hyphae culture medium after a different amount of CFS treatment. **(E)** Nucleic acid content as measured by A_260_ in hyphae culture medium after different amounts of CFS treatment. Vertical bars represent standard deviations of the means. Treatments followed by different letters are statistically different by the Duncan’s multiple range (*p* < 0.05).

Rough hyphae were observed in the CFS treatment group (Figure 5), suggesting that CFS of A4 may damage the cell membrane of hyphae. To test this hypothesis, the cell membrane integrity indicator propidium iodide (PI) was adopted to stain hyphae treated with different concentrations of CFS. The damaged cell membrane is susceptible to dying with PI and gives red fluorescence under the microscope. The results have shown that fluorescence intensity increased in a dose-dependent manner, suggesting an increased degree of damage to the hyphal membrane (Figures 6A,B). Therefore, we detected the malondialdehyde (MDA) content, which is a product of lipid peroxidation damage in the hyphal membrane. The significantly increased MDA content was detected upon CFS presence at a volume fraction of 4% and 6% (Figure 6C). The peroxidation-damaged cell membrane may lead to exudation of cell contents, and the protein and nucleic acid concentration in the hyphal culture medium with or without CFS were further detected. The protein concentration and nucleic acid content increased conspicuously depending on CFS concentration (Figures 6D,E). The CFS of A4 destroyed the integrity of the hyphal membrane of *B. cinerea* by promoting the realization of hyphae membrane peroxidation damage.

### The cell-free supernatant induces reactive oxygen species accumulation in hyphal cells of *Botrytis cinerea*

**FIGURE 7:**
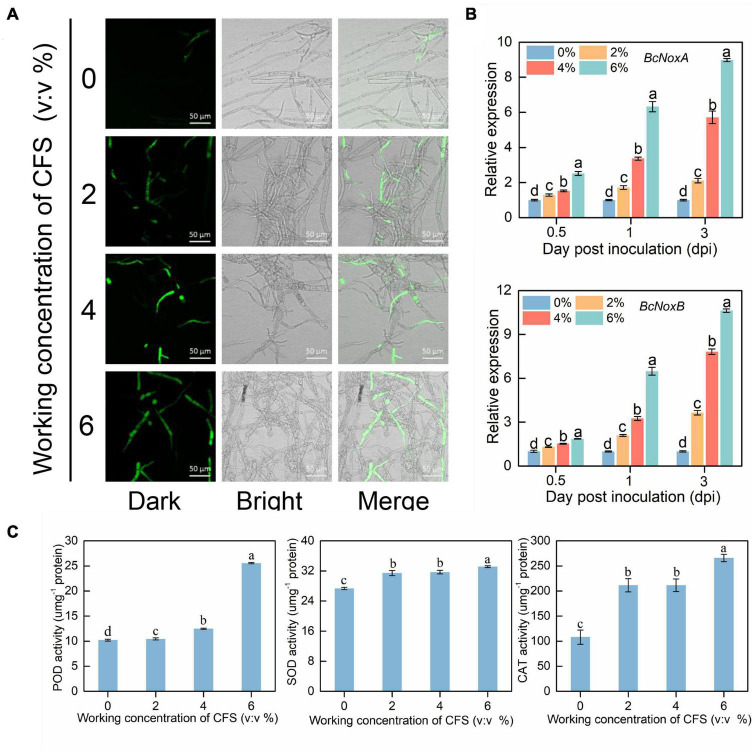
Effects of cell-free supernatant (CFS) on reactive oxygen species (ROS) accumulation in *Botrytis cinerea*. **(A)** The images were recorded using confocal laser microscopy at 488 nm excitation and 520 nm emission. The hyphal cells treated with different concentrations of CFS were harvested after 24 h. Harvested hyphal cells were stained with the oxidant-sensitive probe, H_2_DCFDA, and then washed by PBS buffer for observation. **(B)** Gene expression levels of *NoxA* and *NoxB* at 0.5, 1 and 3 h after treatment**. (C)** The activities of ROS removal-related enzymes (peroxidase, superoxide dismutase and catalase) were recorded in harvested hyphal cells. Different letters above the columns indicate significant differences within each group according to Duncan’s multiple range test (*p* < 0.05).

To further explore the mechanism of hyphal injury caused by CFS of A4, we focused on the change of reactive oxygen species (ROS) in hyphal cells after CFS treatment. We took advantage of H_2_DCFDA, a cell-permeable fluorescence probe, to detect intracellular ROS. As shown in Figure 7A, The accumulation of ROS green fluorescence in hyphal cells increased as the CFS concentration increased, indicating that ROS accumulated continuously after CFS broth treatment. Further, we found that *NoxA* and *NoxB* genes related to ROS synthesis were significantly up-regulated in the very early stage of CFS treatment, and both genes’ expression level was positively correlated with the concentration of CFS treatment (Figure 7B). The accumulation of ROS in hyphal cells usually accompanies the enhancement of scavenging capacity. The enzymatic scavenging pathway of ROS is mainly completed by the sequential cooperation of peroxidase (POD), superoxide dismutase (SOD) and catalase (CAT), which catalyze ROS into harmless substances. We found that the activities of all three enzymes increased significantly after CFS treatment. However, there was no significant dose-dependent effect on the activities of other enzymes except pod (Figure 7C). Taken together, these observations suggest that CFS treatment resulted in oxidative damage of hyphae by inducing the expression of critical genes of ROS formation.

### N-acetylcysteine reduces the accumulation of reactive oxygen species and recovers the weak growth of *Botrytis cinerea* caused by cell-free supernatant

**FIGURE 8:**
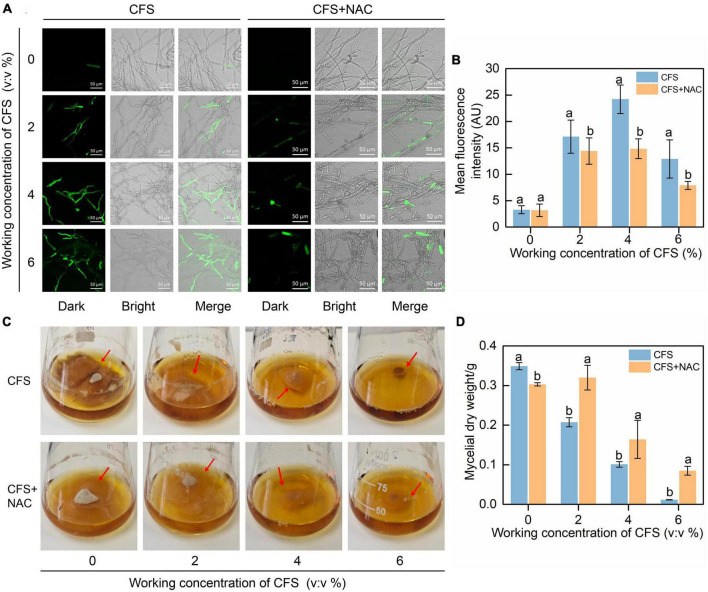
Effect of N-Acetylcysteine (NAC) on the reactive oxygen species (ROS) accumulation and the growth of mycelia treated with cell-free supernatant (CFS). The culture was amended with different concentrations of CFS with or without 10 mM NAC. **(A)** The images were recorded using confocal laser microscopy at 488 nm excitation and 520 nm emission. **(B)** The relative intensity of fluorescence of each treatment was measured by ImageJ. **(C)** Images were recorded after 3 days of incubation. **(D)** Statistical analysis for mycelial dry weight. Error bars indicate standard errors of the means of two repeated experiments. Different letters above the bars indicate significant differences within each group according to Duncan’s multiple range test (*p* < 0.05).

N-acetylcysteine (NAC) is a broad-spectrum inhibitor of ROS, which can effectively remove various types of ROS. We introduced NAC into the hyphae treated with CFS to confirm that the CFS caused *B. cinerea* hyphae damage and inhibition due to oxidative accumulation. The results proved our hypothesis that NAC significantly reduced the CFS-induced increase of green fluorescence signal (Figures 8A,B). In addition, the mycelial ball size and dry weight of *B. cinerea* in PDB increased after NAC was applied (Figures 8C,D). The result confirmed from a different angle that CFS of A4 could induce oxidative damage to the hyphae by inducing a large amount of ROS biosynthesis, thus reducing its growth and pathogenicity.

## Discussion

Gray mold caused by *Botrytis cinerea* has become an economically significant disease in fruit storage conditions, thus garnering interest from post-harvest disease control experts (Zhu and Zhang, 2016; Sun et al., 2019; Sernaite et al., 2020). Plenty of synthetic fungicides have been used for the control of gray mold; due to their continuous and excessive usage, increased resistance has been observed among the pathogens (Jacometti et al., 2010; Yoon et al., 2013; Wang et al., 2021a). Safe and effective disease control methods are the need of the hour. In the past few decades, biocontrol agent *Bacillus* effective against various plant pathogens have been successfully developed and commercialized (Simone et al., 2020). Presently, some of the dominant strains of *Bacillus*, such as *Bacillus velezensis*, have been applied as biological pesticides for the control of gray mold, powdery mildew, and late blight (Lim et al., 2017; Jiang et al., 2018). However, its potential role and mechanism in disease control research are not clarified.

In this study, *B. velezensis* strain A4 was isolated from the surface of kiwifruit and found that its CFS had a significant inhibitory effect on pathogen *B. cinerea* mycelial growth and gray mold in postharvest fruit (Figures 1, 2). CFS of *Bacillus* contains various antifungal components, mainly including antifungal proteins and lipopeptides antibiotics (Sutyak et al., 2008; Torres et al., 2015). These antifungal components resist the invasion of pathogens through multiple mechanisms, including affecting the activity of spores, causing damage to pathogenic hyphae, and enhancing resistance activities of the host (Yanez-Mendizabal and Falconi, 2021). Our results demonstrated that 2–6% CFS of *B. velezensis* A4 could inhibit spore germination of *B. cinerea* at different levels (Figure 3). Previously research proved that *B. velezensis* CE 100 can produce antifungal tetrapeptides, which caused spore germination inhibition and mycelial growth inhibition. This result suggested that CFS of *B. velezensis* A4 might secrete similar components in the role of biological control, and future studies should analysis and isolate antifungal components from it.

Under the CFS stress of A4, the penetrating ability of *B. cinerea* hyphae to onion peel and tomato fruit exhibited reduction (Figure 4), and the hyphae showed abnormal morphology with swelling, rough surface, and breaking in the middle of hyphae (Figure 5), which had been reported in other fungi response to biocontrol bacteria (Liu et al., 2019). Penetration and morphology properties of a pathogen are primarily in determining its pathogenicity. However, little knowledge is exclusive regarding changes in pathogens at the molecular level in response to *B. velezensis* or its bioactive substances. In order to understand the responses of *B. cinerea* under the stress of *B. velezensis* A4, qRT-PCR analysis of growth and pathogenicity regulating genes in *B. cinerea* under CFS of A4 stress was performed, and a total of six related genes were detected and unexceptionally downregulated at varying degrees (Figure 4C). *B. cinerea* intact host plants and form appressoria are required for a successful penetration; this process involves an arrest of polarized growth, apex swelling, and its cell wall reinforcement and is controlled by the *BcPLS1* gene (Gourgues et al., 2004). The inhibition of *BcPLS1* expression induced by CFS stress is likely to be one of the main molecular mechanisms of mycelial *B. cinerea* penetration decline in our work (Figure 4). *BcPG1* and *BcPME1* encode enzymes that hydrolyze pectin, destroy the integrity of plant cells and provide nutrients for the growth and development of *B. cinerea* (Valette-Collet et al., 2003). In comparison, the main contribution of the xylanase *Xyn11A* to the infection process of *B. cinerea* is to induce necrosis of the infected plant tissue (Noda et al., 2010). These functions are in agreement with the observed phenotype on inhibition of mycelia growth and reduction of fruit decay after application of *B. velezensis* A4 CFS (Figures 1, 2). The function of *BcElp4* in *B. cinerea* was investigated by the deletion mutant of *BcElp4*, a *Bcelp4* mutant was significantly impaired in vegetative growth, sclerotia formation and melanin biosynthesis (Shao et al., 2017). Hyphal melanization is a critical pathogenic factor of plenty pathogenic fungi, the absence of melanin will cause the decline or loss of pathogenicity (Francisco et al., 2020). In our study, the expression of *BcElp4* was decreased under the treatment of biocontrol agent A4 and fulfilled the mycelia status in Figure 1 that control mycelia accumulated more melanin than that of the treatment group.

During the interaction between *B. cinerea* and *B. velezensis* A4, some antifungal substances secreted from A4 enter into *B. cinerea* cells to inhibit gene expressions and lead to abnormal hyphal morphology. The enhanced fluorescence intensity of hyphal after CFS of A4 treatment indicated that the hyphae cell membrane was significantly damaged (Figure 6). Meanwhile, *B. cinerea* may cope with these antifungal substances by activating reactive oxygen species (ROS) synthesis, which may function as a singling molecule of external stress respond in *B. cinerea.* It is understood that the accumulation of excessive ROS in the cells may have adverse effects on fungal cell membranes, resulting in growth retardation (Li et al., 2019b; Cui et al., 2021; Sun et al., 2021). Treatment with CFS of A4 upregulates the expression of ROS-produced genes in hyphal cells of *B. cinerea*, and ROS scavenging enzymes are simultaneously activated (Figure 7). *Bacillus* sp. AF-1 has also been reported to increase ROS accumulation in pathogen to jeopardize its virulence, but not detected the ROS production genes and eliminated enzymes (Xuan et al., 2017). In addition, ROS scavenger N-acetylcyseteine (NAC) could significantly attenuate CFS-induced ROS accumulation, making its level close to the untreated state (Figures 8A,B) and alleviating the limitation of CFS on the growth of mycelia *B. cinerea* in medium (Figures 8C,D). The above results indicate that the hyphal cells of *B. cinerea* are forced to accumulate excessive reactive oxygen species by A4-induced gene expression treatment, thereby impairing cells and further affecting their typical growth and pathogenicity on the host.

## Conclusion

Results showed that hyphal growth, spore germination rate, and hyphae penetration ability of *B. cinerea* were markedly inhibited by the CFS of *B. velezensis* A4. Moreover, the CFS effectively reduced the lesion diameter of cherry tomato, apple, kiwifruit, and strawberry caused by *B. cinerea*. Scanning electron microscopy observed seriously damaged hyphae with A4 CFS treatment, and the damaging effect was mainly caused by the accumulation of excessive reactive oxygen species (ROS) in *B. cinerea* hypha. Excessive ROS brings membrane peroxidation, impairs membrane integrity, and causes the leakage of cell constituents, resulting in reduced hyphal growth and virulence. We conclude that the CFS of *B. velezensis* A4 might has a great potential in controlling postharvest gray mold during postharvest storage.

## Data availability statement

The original contributions presented in this study are included in the article/supplementary material, further inquiries can be directed to the corresponding author.

## Author contributions

HZ performed the experiments and wrote the manuscript with the assistance of KL. JC, HL, YF, and WS participated in the data curation and analysis. MM designed the research and provided financial support. MM and YL revised the manuscript. All authors have read and approved the submitted version of the article.
